# Profile of otorhinolaryngology emergency unit care in a high complexity public hospital

**DOI:** 10.5935/1808-8694.20130056

**Published:** 2015-10-04

**Authors:** José Santos Cruz de Andrade, André Maranhão Souza de Albuquerque, Rafaella Caruso Matos, Valéria Romero Godofredo, Norma de Oliveira Penido

**Affiliations:** a Resident physician - Department of Otorhinolaryngology and Head and Neck Surgery - Federal University of São Paulo - Paulista School of Medicine.; b MSc student - Graduate Program - Department of Otorhinolaryngology and Head and Neck Surgery - Federal University of São Paulo - Paulista School of Medicine. Attending ENT.; c Medical Student - Federal University of São Paulo - Paulista School of Medicine.; d Affiliated Teacher - Department of Otorhinolaryngology and Head and Neck Surgery - Federal University of São Paulo - Paulista School of Medicine.

**Keywords:** emergencies, epidemiology, otolaryngology

## Abstract

Urgent and emergency care are common happenings in ENT practice and most carry low morbidity and mortality. There are but few studies that address the epidemiology of these situations.

**Objective:**

To evaluate the epidemiological characteristics of care in the emergency department of otorhinolaryngology at a high complexity hospital.

**Method:**

Epidemiological, cross-sectional study, retrospective with data collection carried out from medical records from the emergency department of otorhinolaryngology of a high complexity hospital in São Paulo, for a period of 12 months. Data collected: age, gender, clinical diagnosis and management. The cases were divided by subspecialty: otology, rhinology, pharyngolaryngealstomatology and head and neck surgery. We evaluated the level of urgency/emergency, etiology and monthly distribution of visits.

**Results:**

17,503 medical records were obtained; 1,863 were excluded. Of the 15,640 cases included, the average age was 36.3 years. 9,818 (62.77%) corresponded to cases considered as emergency/ urgency. Among the urgency/emergency cases, 6,422 (65.41%) were diagnosed in the ear and among the 10 most prevalent diagnostics, 7 were in the subspecialty of otology.

**Conclusion:**

Among the patients seen in the emergency department of otolaryngology evaluated in this study, 62.77% corresponded to cases of urgency/emergency, predominantly in the otology subspecialty.

## INTRODUCTION

Urgent/emergency care in ENT is a common situation, and most represent low morbidity/mortality, with a clear predominance for infectious/inflammatory disorders^1^. However, potentially lethal ENT disorders do exist, and the need for prompt intervention is paramount^1^, ^2^, ^3^.

The city of São Paulo has an estimated population of 10,886,518 inhabitants^4^ and the largest public hospitals network in Brazil. The hospital where the study was carried out is a high complexity tertiary healthcare center, predominantly caring for the resident population of south São Paulo, besides being a reference for the other regions of the state and the country^4^, ^5^.

Just a handful of the care provided in emergency ENT services corresponds to true emergencies. Amongst these emergencies, an even smaller number require immediate surgical intervention. There is an argument in the literature regarding the real need for an ENT emergency care as a non-referenced unit, working as a walk-in clinic. Nevertheless, our specialty has an essential role to play in common disorders in different age ranges, such as severe epistaxis and aerodigestive foreign bodies, among other potentially lethal disorders^2^, ^3^, ^6^, ^7^. In recent years, there has been an increase in the number of patients seen in emergency/urgency services, and associated to this phenomenon, amongst other factors, there is the difficulty of access to medical specialties, long waiting queues and misinformation regarding the healthcare system^2^, ^3^.

This study aimed at assessing the epidemiological (age, gender) and etiological aspects, specialty subdivision, the need for hospitalization, surgical intervention and the monthly distribution of all emergency ENT care provided in a high complexity hospital in the city of São Paulo.

## METHOD

This is an epidemiological, cross-sectional study carried out in the ENT emergency ward of the city of São Paulo, encompassing the time period between february 1^st^, 2010 through january 31^st^ of 2011, with data collection from the digitalized patient charts, and the following items were considered: age, gender, clinical diagnosis and behavior.

The medical care was provided by the attending otorhinolaryngology and the ENT or head and neck surgery resident - working full time. The inclusion criteria included all the patients seen at the ENT emergency ward. The exclusion criteria were: non-ENT-related diseases, returns, non-digitalized charts and those with incomplete data.

The care was broken down into subspecialties, based on clinical diagnosis: otology, rhinology, pharyngolaryngo-stomatology and head and neck surgery (HNS). We computed one diagnosis per visit, considering the patient's main complaint. The events were broken down into: urgency/emergency and not-urgency and not-emergency (not urgency/emergency) from the clinical diagnosis, taking into consideration the Cuchi's etiological classification^6^ and the patient's clinical condition, being subdivided into: inflammatory/infectious events, traumas, bleeds, foreign bodies, tumors, functional disorders, neurosensory problems, respiratory and unclassified diseases.

The urgency/emergency cases were also assessed as to their monthly distribution. The following diagnoses were considered as urgency/emergency:

• In otology: auricular abscess/infected coloboma, foreign body, herpes zoster, ear myiasis, bullous myringitis, external otitis (EO), acute otitis media (AOM), flared-up chronic otitis media (COM), mastoiditis, peripheral facial paralysis (PFP), perichondritis, vestibular syndrome, sudden hearing loss, ear trauma
• In rhinology: nasal abscess, facial cellulitis, foreign body, dacryocistitis, epistaxis, septal hematoma, acute rhinosinusitis, complicated rhinosinusitis, nasal trauma, ear vestibulitis
• In pharyngolaryngo-stomatology: peritonsillar abscess, aphthae, foreign bodies, pharyngotonsillitis, hemorrhage, acute laryngitis, temporomandibular (TMJ) joint dislocation, oral myiasis, sialadenitis, trauma
• In HNS: neck abscess, tumor-causing dyspnea, tumor-related pain, laryngeal/tracheal stenosis, tumor-related hemorrhage, lymphadenitis, parotiditis, submandibular disorders.

The following diagnoses were not considered as urgency/emergency:

• In otology: ear wax, TMJ dysfunction, Eustachian tube dysfunction, hearing loss, non-flared up COM, removal of dressings/stitches, non-flared up ear vestibular syndrome, tinnitus, others
• In rhinology: non-flared up chronic rhinosinusitis, nasal polyposis, nasal tumors, common cold, allergic rhinitis, nasal packing removal, others
• In pharyngolaryngo-stomatology: chronic tonsillitis, non-acute dysphagia, oral candidiasis, oral cavity tumors, others
• In HNS: goiter, lymph node enlargement, undetermined neck mass, dressing removal, not-flared up sialolithiasis, elective exchange of a tracheostomy cannula, others.

Besides the ENT care, we computed all the emergency room procedures in different specialties: general surgery, general practice, gynecology/obstetrics, psychiatry, neurology, neurosurgery, ophthalmology, orthopedics and pediatrics.

This study was submitted and approved by the Ethics in Research Committee, number 0081/10. The data was stored and analyzed in the Excel 2008^®^ software.

## RESULTS

In the emergency room of the hospital where the study was carried out, 205,486 patients were cared for between february 1^st^ and january 31^st^, 2011. 18,279 (8.89%) were patients seen in the ENT emergency department. Table 1 depicts the number of patients seen by specialty within the period of the study.

**Table 1:** Number of complaints divided by specialty.

Specialty	Number of patients seen
Pediatrics	26,075
Otolaryngology	17,503
Orthopedics	26,936
Ophthalmology	50,395
Neurology	6,793
Neurosurgery	1,536
Psychiatry	3,924
Gynecology/Obstetrics	12,337
General practice	37,361
General surgery	21,851
Total	204,701

In the period established for the study, we gathered 17,503 charts from patients in the ENT emergency room. 1,863 charts were taken off the study because of: 607 patients did not answered our call (3.44%), 523 were return patients (2.98%), 434 cases were not otorhinolaryngology disorders (2.47%) and 299 charts were taken off because of inadequate filling out (1.70%). We had a total of 15,640 charts included in the study.

Among the charts included, the mean age was 36.3 years, with a median value of 37 years. As far as age is concerned, 25.48% of the patients had between 0 and 15 years; 66.74% had between 16 and 65 years and 7.78% were older than 66 years. As far as gender was concerned, 8,523 (54.49%) were females, and 7,117 (45.50%) individuals were males.

Among the 15,640 charts included in the study, 9,818 (62.77%) corresponded to urgency/emergency care and 5,822 (37.22%) patients were not considered as requiring urgency or emergency care.

Table 2 depicts the division among subspecialties of the care considered urgency/emergency.

**Table 2:** Division by subspecialties among the patients considered urgency/emergency care.

Subspecialty	
Otology	6,422 (65.41%)
Rhinology	1,767 (17.99%)
Pharyngolaryngo-stomatology	1,453 (14.79%)
Head and neck surgery	176 (1.79%)
Total	9,818 (100%)

Table 3 depicts the etiological subdivision of the urgency/emergency care provided, following the criteria described by Cuchi^6^.

**Table 3:** Etiological subdivision among the urgency/emergency care provided.

Etiology	
Inflammation/Infection	6,386 (65.04%)
Sensorineural disorders	1,342 (13.66%)
Foreign bodies	960 (9.77%)
Hemorrhages	657 (6,69%)
Trauma	439 (4.47%)
Tumor disorders	24 (0.24%)
Functional disorders	6 (0.06%)
Respiratory disorders	4 (0.04%)
Not classified	0 (0%)
Total	9,818 (100%)

**Figure 1:**
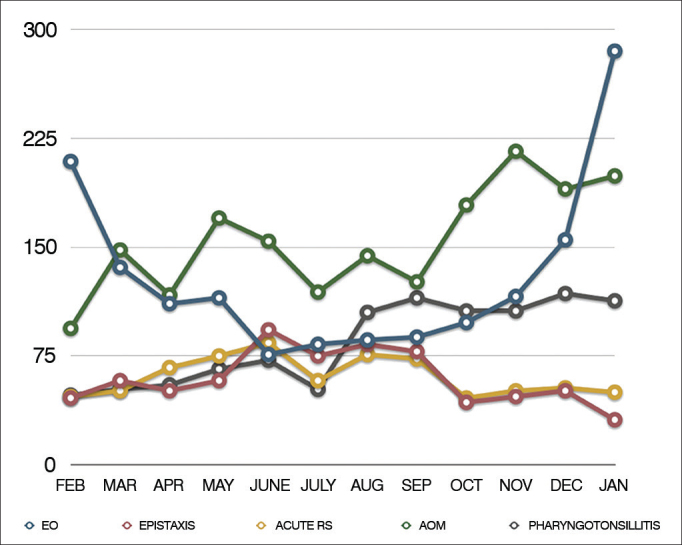
Monthly distribution of fve amongst the ten most prevalent diagnoses in the urgency/emergency care provided. EO: External otitis; AOM: Acute otitis media.

Among the urgency/emergency care provided, we carried out a division associated with the months of the year. The ten more prevalent diagnoses among the urgency/emergency care provided are displayed on Table 4, and Figure 1 shows the monthly distribution of five of these diagnoses.

**Table 4:** List of the ten most prevalent diagnoses among the urgency/emergency care.

Diagnosis	Number of patients seen
Acute otitis media	1,856
External otitis	1,558
Pharyngotonsillitis	1,008
Vestibular syndrome	739
Acute Rhinosinusitis	711
Foreign body in the ear	666
Epistaxis	642
Flared up chronic otitis media	604
Peripheral facial paralysis	466
Ear trauma	291

Among all the patients included in the study (urgency/emergency + not urgency/emergency = 15,640), only 168 (1.07%) needed hospitalization, and 81 (0.51%) required surgery.

## DISCUSSION

The ENT urgency/emergency department is responsible for a large share of the care given in high complexity hospitals^7^. We noticed, however, that among the services provided, a significant share represents non-urgency/ non-emergency care, diseases not associated with ENT and disorders which should be seen by the general practitioner^7^, ^8^. In our study, 5,822 patients did not require urgency/emergency care. These 5,822 cases, corresponding to 37.22% of the charts included in the study, incur expenses, cause reduction in service quality and efficiency, thus impairing urgency/emergency care.

Law 8080/90 of the Brazilian Constitution, which governs the healthcare system, establishes that healthcare must be provided in a hierarchic way, in growing levels of complexity. This way, in elective clinical situations, the patient must follow a referral and counter-referral flow from the primary to the quaternary level, according to patients’ needs. However, we noticed that the ENT ER is used as an alternative to specialized outpatient clinics which are difficult to reach^3^, ^6^, ^8^.

There are just a few papers describing the epidemiology in ENT care and there is a heterogeneity of reports vis-à-vis the classification methods used in this care^8^. These methods vary as to their definition of urgency and emergency and as to the subdivision of the subspecialties, besides differences as to the routines of each service. It is clear that the more rigorous the classification criteria used to define urgency and emergency, the lower the prevalence of these situations in studies. It is, however, noteworthy that non-urgency/emergency cases make up an excessive fraction of the services provided. Amongst the evaluated studies, there are varied percentages of urgency/ emergency care: in the study carried out by Furtado et al., of 2011^8^, we found similar percentage values of: cases classified as urgency/emergency (61.26%), distribution by gender (54.48% of females and 45.51% of males), division by subspecialties (predominance of Otology) and infectious/inflammatory etiology.

Despite these similarities, there is a limitation vis-à-vis the comparison of this data, because our study deals with a different population from another type of healthcare service. The city of São Paulo boasts a better distribution of healthcare, with more options for the population, including primary and secondary care. Other studies show smaller percentages of ENT urgency/emergency care in different modes of classification. Timsit et al.^7^ considered that only 10% of the cases seen were urgencies that demanded ready intervention^1^, ^2^, ^6^, ^7^, ^8^. Otology was the area with the highest percentage of urgencies/emergencies, with 6,422 (65.41%), in agreement with other studies, stressing the importance of the subspecialty within Otorhinolaryngology and the particularities of its semiology^2^, ^8^.

It is worth stressing as advantages of this study, the high number of patients seen and the fact that this emergency room cares for the cases of head and neck surgery as well, which is not universal among ENT services - where these patients are treated by general surgery. As limitation to the interpretation of our data, 1,863 patient charts were taken off our study, a larger number when compared to other similar studies^6^, ^7^, ^8^.

When broken down into months, almost all diagnoses were evenly distributed over the months of the year. Seasonality was observed with regards to external otitis, more prevalent in the summer months, classical feature of the disease already widely described by other authors^9^, ^10^. In comparison with otitis media, which showed a constant prevalence, external otitis is a less frequent diagnosis in all months of the year, with the exception of january and february, when this frequency is reversed. In these summer months, swimmer's ear is the most common diagnosis among all cases seen.

As far as epistaxis is concerned, we did not notice such a clear seasonality, as expected, although the winter months, june and august, were the months with the highest number of cases (73 and 69 cases, respectively). Studies have associated epistaxis to the dry and cold climate, with weather correlation^11^, ^12^, although there is no consensus in the literature^13^. It is possible that Brazilian climate characteristics, with little defined seasons, could be an explanation for the lack of evident seasonality in the distribution of epistaxis and other diagnoses in our study.

The common cold was classified as a non-urgency/ emergency situation in rhinology, because of its benign outcome, spontaneous resolution of symptoms and the possibility of management by general medical practitioners. Other authors also rated the common cold as not requiring urgency/emergency care^8^, ^14^.

We noticed a small rate of hospitalization and surgical interventions among our emergency room care, and such data is in accordance with other studies^6^, ^7^, ^8^. This data is associated with the characteristics of the specialty, but also the difficulties found in public healthcare services, lack of beds and outpatient treatment of disorders which usually require hospitalization.

Among the ten most frequent complaints, seven belonged to the subgroup of otology, and AOM (acute otitis media) alone represented 11.86% of all complaints included in this study. This data differ from that other studies which found a predominance of care associated with pharyngeal disorders^15^, ^16^, ^17^.

The ENT emergency room plays a key role in the care of life-threatening conditions such as severe epistaxis, acute respiratory failure, post-tonsillectomy bleeding, neck abscess, invasive fungal rhinosinusitis, complications of middle ear infections and malignant external otitis, among others. These are situations requiring immediate evaluation and management by an ENT physician, which can justify the presence of the specialist in emergency rooms in high complexity hospitals. However, it is important to stress that, as an implication of our findings, an ENT emergency room, working as a referral center, may reduce the number of non-urgency/emergency cases. This is a possible alternative, given the low prevalence of care requiring hospitalization and surgical intervention, indicating the low severity of these cases. A general emergency room, with specialized physicians on backup could refer the pertaining cases which require prompt care.

## CONCLUSION

Among the ENT emergency room complaints assessed in this study, 62.77% corresponded to cases of urgency/emergency care, with predominance in the otology subspecialty. Among the urgent/emergency care provided, there was a predominance of inflammatory/ infectious disorders and a low prevalence of cases requiring hospitalization and surgery.
